# Supplementary material for: Implementation of a screening, brief intervention and referral to treatment programme for risky substance use in South African emergency centres: A mixed methods evaluation study
Source: PLoS One. 2019 Nov 15;14(11):e0224951. doi: 10.1371/journal.pone.0224951 (PMC6858052; doi:10.1371/journal.pone.0224951)
Supplement: S1 File — (DOCX) [file pone.0224951.s001.docx]

# Appendix 1: Use of the CFIR

## CFIR constructs operationalized

Within the CFIR framework, we operationalized the ‘outer setting’ as the (i) the geographical location of the hospitals and the communities served by the hospital, (ii) the Premier’s office, and the (iii) provincial offices of the Departments of Health and Social Development (see Fig 1). Within this study, these communities and organisations comprise the context within which the implementing partners worked to introduce and institutionalise the SBIRT programme. The ‘inner setting’ for this study included the emergency centres and hospitals implementing the programme, as well as the district Department of Health offices, the regional Department of Social Development offices and the non-profit organisations (NPOs) which employ and supervise the SBIRT counsellors. Since the district Department of Health offices are responsible for contracting the NPOs, and overseeing the programme operations, they were judged to be within the “structural, political and cultural contexts through which the implementation process will proceed” (Damschroder et al., 2009). Similarly, the regional Department of Social Development offices were included in the inner setting as they were the implementing partner for referrals of patients at high risk of substance-related harms.

**Fig S1. Outer and inner setting**

## CFIR constructs excluded

In the ‘intervention characteristics’ domain, the construct ‘relative advantage’ was not utilised as there is currently no substance use or behaviour change programme offered in the ECs, thus the SBIRT programme could not be compared to any existing programmes. One construct was excluded from the ‘outer setting’ domain, namely ‘cosmopolitanism’ as we decided that this was not applicable in the public health system. From the ‘inner setting’ domain we excluded ‘structural characteristics’ and ‘culture’ as we did not assess this. Additionally, constructs were excluded from the ‘characteristics of individuals’ domain, since the SBIRT programme added new staff to ECs who were responsible for delivering the intervention, and the introduction of the programme required minimal behaviour change from existing staff. Thus, we excluded ‘self-efficacy’ and ‘individual stage of change’. We retained the other constructs as we hypothesized that these factors influenced stakeholders’ attitudes towards the programme and its sustained use. In the ‘process of implementation’ domain, ‘opinion leaders’ was excluded as this construct is applied to stakeholders in the inner setting who have influence with their colleagues regarding implementation of the innovation. In the SBIRT programme, the counsellors delivered the intervention, and the stakeholders involved all had formal roles in the implementation process. ‘Champions’ and ‘external change agents’ were excluded for similar reasons. See Table S1 below for the CFIR constructs applied in this study.

## CFIR constructs included

The CFIR constructs used in this study are tabulated below.

**Table S1. CFIR constructs used by domain**
