# Supplementary material for: Implementation of a screening, brief intervention and referral to treatment programme for risky substance use in South African emergency centres: A mixed methods evaluation study
Source: PLoS One. 2019 Nov 15;14(11):e0224951. doi: 10.1371/journal.pone.0224951 (PMC6858052; doi:10.1371/journal.pone.0224951)
Supplement: S1 Table — (DOCX) [file pone.0224951.s002.docx]

| **Intervention characteristics** | **Outer setting** | **Inner setting** | **Characteristics of individuals** | **Process of implementation** |
| --- | --- | --- | --- | --- |
| Intervention source | Needs & resources of those served by the organisation | Networks & communications | Knowledge & beliefs about intervention | Planning |
| Evidence strength & quality | Peer pressure | Implementation climate:   - *Tension for change* - *Compatibility* - *Relative priority* - *Organisational incentives & rewards* - *Goals & feedback* - *Learning climate* | Other personal attributes | Engaging   - *Formally appointed internal implementation leaders* - *Key stakeholders* - *Innovation participants* |
| Adaptability | External policy & incentives | Readiness for implementation (explored retrospectively)   - *Leadership engagement* - *Available resources* - *Access to knowledge & information* |  | Executing |
| Complexity |  |  |  | Reflecting & evaluating |
| Design quality & packaging |  |  |  |  |
