# Supplementary figures and images for: Implementation of a screening, brief intervention and referral to treatment programme for risky substance use in South African emergency centres: A mixed methods evaluation study
Source: PLoS One. 2019 Nov 15;14(11):e0224951. doi: 10.1371/journal.pone.0224951 (PMC6858052; doi:10.1371/journal.pone.0224951)

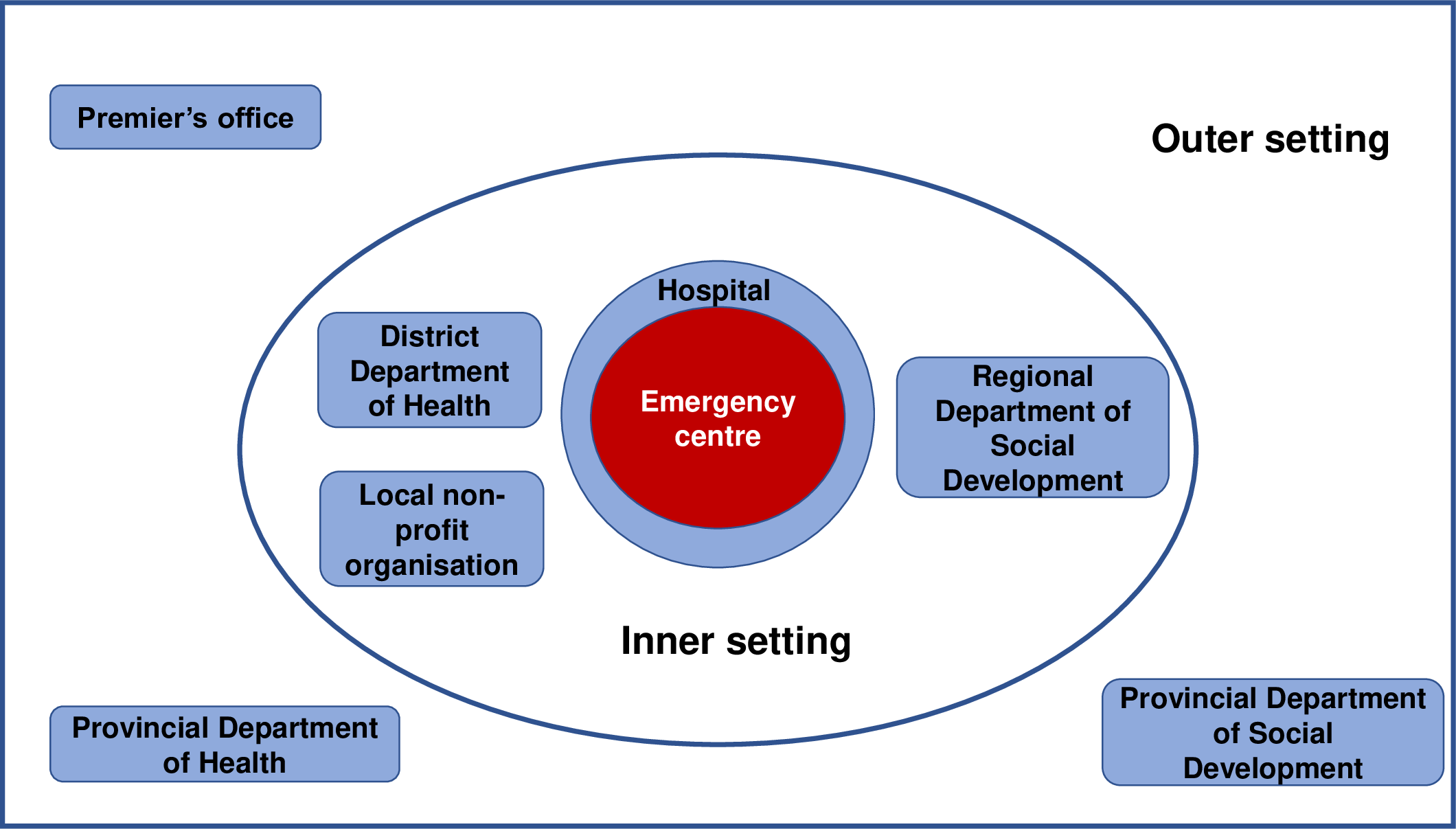

Supplement: S1 Fig — (TIF) [file pone.0224951.s003.tif]
